# Impact of online health education on attention-deficit/hyperactivity disorder screening results and parenting stress among school-aged children

**DOI:** 10.3389/fpsyt.2025.1522263

**Published:** 2025-01-31

**Authors:** Jing Tan, Wenxia Yi, Jianna Shen, Bin Peng, Min Gong, Feng Li, Li Chen

**Affiliations:** ^1^ Growth, Development and Mental Health Center of Children and Adolescents, Chongqing Key Laboratory of Child Neurodevelopment and Cognitive Disorders, National Clinical Research Center for Child Health and Disorders, Ministry of Education Key Laboratory of Child Development and Disorders, Children’s Hospital of Chongqing Medical University, Chongqing, China; ^2^ Institute of Basic Education and Teaching, Chongqing Educational Science Research Academy, Chongqing, China; ^3^ School of Public Health, Chongqing Medical University, Chongqing, China; ^4^ Department of Pediatrics, Richmond University Medical Center, New York, NY, United States; ^5^ Department of Pediatrics, Jiangjin Centre Hospital, Chongqing, China

**Keywords:** attention-deficit/hyperactivity disorder (ADHD), online health education lecture, health communication, positive screening rate, parenting stress

## Abstract

**Aims:**

To investigate the effects of an online health education lecture on the positive screening rate of attention-deficit/hyperactivity disorder (ADHD) and parenting stress among parents of children diagnosed with or screened positive for ADHD.

**Methods:**

Using stratified proportional random cluster sampling, 14 primary schools in Chongqing were selected to conduct an online lecture about ADHD for parents and teachers. A total of 2,611 questionnaires were collected (1,508 intervention group, 1,103 control group).

**Results:**

The lecture did not significantly affect the positive screening rate of ADHD (parents: β=-0.37, p=0.208; teachers: β=0.53, p=0.338); however, the positive screening rate increased post-intervention. Inattention scores were higher in the intervention group (β=0.42, p=0.040). Parents as primary caregivers were associated with lower ADHD symptom scores (β=-0.61, p=0.022). Lower parental education levels were associated with higher ADHD screening rates (β=0.49, p=0.039) and symptom scores (β=0.60, p=0.022). Teachers with 10-19 years of experience had higher positive screening rates (β=1.26, p=0.005) and symptom scores (β=2.60, p<0.001). The intervention did not affect parenting stress (Z=-1.413, p=0.158).

**Conclusions:**

The lecture’s effects were relatively weak, using questionnaires may have facilitated health communication. Individual characteristics of parents and teachers should be considered in assessments (ClinicalTrial.gov ID: NCT05231902).

## 1 Introduction

Attention-deficit/hyperactivity disorder (ADHD) is one of the most common chronic neurodevelopmental disorders in children; it is characterized by core symptoms of inattention, hyperactivity, and impulsivity that are inconsistent with developmental levels. ADHD is prevalent in 2.2% to 7.2% of the population at the community level. Globally, the average prevalence is approximately 5% (1). China has a higher prevalence of ADHD at 6.3% (2), and the incidence is high among school-aged children. Despite this, the rate of seeking medical attention for ADHD remains relatively low (3). As the most common developmental behavioral disorder, the recognition of ADHD relies heavily on the observation of parents or teachers. However, a study has shown that the general public generally does not have a good understanding of ADHD (4). This may result parents failing to detect abnormalities early, consequently reducing the rate of seeking medical attention for ADHD. Previous analyses of European literature on ADHD incidence and diagnosis over the past decade have shown a significant gap between the onset and diagnosis of ADHD in children. While the age of onset for children with ADHD alone ranges from 2.25 years to 7.5 years, the age of diagnosis ranges from 6.2 years to 18.1 years (5). Furthermore, delayed detection can affect the family’s satisfaction with diagnosis and treatment. A retrospective study from France reported a satisfaction rate of only 61% after initial referral among families with ADHD. The most common reason for dissatisfaction was the significant time gap between diagnosis and treatment, with inadequate early identification of ADHD by school professionals being a contributing factor (6). This finding is consistent with the results of a questionnaire survey conducted among 636 teachers, which showed that only 44.8% of them had a good understanding of ADHD, although 84.1% held positive attitudes toward it. (7). Therefore, improving parents’ and teachers’ knowledge of ADHD may contribute to its early diagnosis in children. ADHD not only affects various aspects of the physical health, learning, socialization, and occupational functioning of affected children (8), but also has a negative impact on their families. Parents of children with ADHD often report higher levels of parenting stress than parents of typically developing children, which may be related to the core symptoms and functional impairments of ADHD. The inattention, hyperactivity, and impulsivity behaviors exhibited by children with ADHD can exacerbate parenting stress, which leads to parents adopting stricter and authoritarian parenting styles. This in turn negatively affects children’s emotional regulation, social interaction skills, and adaptability (9, 10) that if left unaddressed can create a vicious circle. Previous research has shown that providing ADHD-related information and parental training can increase parental knowledge, positive attitudes, and positive behaviors toward children with ADHD (11).

Health communication refers to the process of exchanging and sharing information and emotions to promote health. Research on health communication in the field of ADHD has primarily focused on children diagnosed with ADHD and their families (11–14). Surveys targeting the general public have mostly been limited to assessing knowledge levels and attitudes toward the disorder (4, 7, 11). An important question is whether health education for the general public can enhance awareness of ADHD, leading to earlier diagnosis and treatment for children with the disorder. Additionally, for parents of children who screen positive for ADHD, we are interested in whether scientific knowledge communication can alleviate parenting stress. In this study, “positive screening for ADHD” (15) is defined as a positive result based on the Vanderbilt ADHD Diagnostic Parent/Teacher Rating Scale (VADPRS/VADTRS). It is important to note that a positive screening result does not equate to a confirmed diagnosis but indicates the need for further evaluation in a clinical setting. The final diagnosis of ADHD must be determined by a clinician. To address these questions, we designed a cluster randomized controlled trial (RCT) to conduct an online health education lecture for parents and teachers from multiple schools in Chongqing, China. We hypothesize that (1) health education for the general public can increase the positive screening rate for ADHD, and (2) online health education can reduce parenting stress among parents of children who screen positive for ADHD.

## 2 Method

### 2.1 Study design and setting

This cluster RCT targeted parents and teachers of children aged 6-12 years. The study was conducted in 15 (14 for round 2) primary schools between October 2021 and May 2022 in Chongqing, China. The survey was done online; the parents or teachers had the right to refuse participation. Teachers having served as the homeroom teacher for the participating child’s class for at least 1 month were included. Parents having a child aged 6-12 years attending the primary school were included. Parents or teachers who did not understand the content of the study or refused to participate were excluded.

This study employed stratified proportional random cluster sampling. The total number of primary school students in four functional regions (central urban area, main urban district, northeastern district, and southeastern district) of Chongqing was determined, and the proportion of students in each region was calculated. A total of 15 schools were randomly selected, and within each school, 2-3 classes from grades to 2 to 5 were randomly chosen, with the class serving as the cluster unit [for more detailed sampling information, refer to the preliminary research conducted by our research team (16)]. To ensure participant anonymity, each school, grade, and class was assigned a unique code by the researchers, with the last two digits serving as each child’s personal identification code, assigned by the teacher. Parents and teachers were instructed to use only this code when completing the questionnaires, ensuring the anonymity of their responses. Parents and teachers completed the first round of questionnaires.

Considering that the mode of intervention was online, it was possible for parents or teachers who were in the same geographical area to become acquainted with and privately share the lecture QR code. Therefore, the schools were randomly divided into the intervention and control groups based on their functional regions. The randomization sequence was generated by a researcher using R version 4.0.5 (R Foundation for Statistical Computing, Vienna, Austria). As one school dropped out of the study due to low teacher participation, 14 schools were included in Round 2, with six schools in the intervention group and eight schools in the control group. Five months after completing the first round of questionnaires, all parents and teachers in the intervention group received a 90-minute online health education lecture. One month after the lecture, the parents and teachers completed the second round of questionnaires separately.

No additional interventions were provided to the control group before they completed the second round of questionnaires. The parental questionnaire included basic information, the Chinese validated versions of VADPRS and Caregiver Strain Questionnaire (CGSQ). The teacher questionnaire included basic information and the Chinese validated version of VADTRS. The parents, teachers, and pediatricians were aware of their allocated arms. A researcher blinded to the group allocation conducted the primary and secondary analyses. This trial was registered on Clinicaltrial.gov, NCT05231902.

### 2.2 Interventions

The intervention group received a 90-minute comprehensive online lecture on ADHD. The parent lecture focused on the following topics: What is wrong with my child? What are the effects of ADHD in children? What causes ADHD? How can ADHD be identified and diagnosed? How should ADHD be treated? Frequently asked questions were also addressed. The teacher lecture mainly covered the following topics: What is ADHD? What causes ADHD? How can ADHD be identified and diagnosed? How is ADHD managed in the Classroom?.

All lectures were delivered by a senior developmental-behavioral pediatrician and a senior psychiatrist and used the same version of PowerPoint slides for the parents and teachers. To ensure fairness for the control group, the same lecture content was provided to parents and teachers in the control group after completion of the second round of questionnaires.

### 2.3 Quality control

To maintain quality control, we repeated the third question of the Vanderbilt questionnaire. Specifically, parents and teachers assessed the inattention question “Does not seem to listen when spoken to directly” twice in each questionnaire. To account for the subjective nature of the responses, a score difference of up to 1 point between the two evaluations was considered acceptable. For example, if the first evaluation was rated as “Never” and the second as “Never” or “Occasionally,” the response was valid. However, if the second evaluation was rated as “Often” or “Very Often,” the response was deemed invalid and excluded from the analysis.

### 2.4 Measures

#### 2.4.1 Primary outcome measures

The Vanderbilt ADHD Diagnostic Parent/Teacher Rating Scale is designed to measure the severity of ADHD symptoms in children aged 6 to 12 years. It comprises two components: symptom assessment and performance impairment. The symptom assessment screens for symptoms relevant to inattentive (items 1-9) and hyperactive (items 10-18) ADHD. Items 36-43 and 49-56 are performance measures. Symptom measures on the scale range from 0 to 3. A positive response in the symptom assessment section is 2 or 3 (often or very often). The performance measures on the scale are scored from 1 to 5, with 4 and 5 indicating problematic. The scoring standard for ADHD is as follows: a score of 2 or 3 on 6 of the 9 items in questions 1-9 and/or questions 10-18 and functional impairment (defined as at least 2 items scoring 4 or at least 1 item scoring 5 in the performance section’s 8 items) (17). Higher scores indicated worse outcomes. The VADPRS’s Cronbach α is 0.906, while that of the VADTRS’s is 0.937 (18). Using the DSM-IV as the reference standard, the VADPRS has an overall sensitivity of 90.2% for ADHD, a specificity of 62.2%, and a diagnostic agreement rate of 72.5% (19).

The CGSQ is used to assess pressure owing to their children’s problems among parents of children aged <18 years. The 21 items of the CGSQ are divided into three dimensions to assess parenting pressure: objective pressure, subjective internal pressure, and subjective external pressure. Each item is graded on a 5-point Likert scale; the total scores range from 21 to 105, and the higher the score, the greater the parenting pressure on caregivers. The internal consistency Cronbach α of the CGSQ is 0.93 (20).

All questionnaires were administered at baseline (i.e., 5 months before the health education lecture) and 1 month after the lecture.

#### 2.4.2 Demographic variables

This study collected data on children’s sex, age, primary caregivers, respondents, respondents’ education level, teachers’ years of teaching experience, and the number of previous ADHD-related training sessions attended by teachers.

#### 2.4.3 Power and sample size

The sample size was calculated using the event per variable (EPV) method. Based on the ADHD positive screening rate calculated from our first round of parent questionnaires, six influencing factors were considered, namely, intervention, sex, age, primary caregivers, education level of respondents, and baseline ADHD symptom scores. A minimum of 60 positive outcomes (6 × 10) were required, assuming a positive screening rate of 3.7% (112/2997) in the first round. Therefore, the minimum required sample size was 1621 (60/0.037).

### 2.5 Statistical analysis

In total, 2611 questionnaires were analyzed, with 1508 questionnaires in the intervention group and 1103 questionnaires in the control group. Owing to the nested data structure, a generalized linear mixed model was used for the primary outcome analysis, considering the hierarchy of region-school-individual. In the analysis of parent questionnaires, the child’s sex, primary caregivers, respondents’ education levels, and scores from the first round of parent questionnaires were included as covariates. In the analysis of teacher questionnaires, the child’s sex, teachers’ years of teaching experience, number of previous ADHD-related training sessions attended by teachers, and scores from the first round of teacher questionnaires were included as covariates. When the outcome variable was binary, a binary logistic regression with a logit link function was used. When the outcome variable was continuous, linear regression with a linear link function was used. Continuous variables were analyzed using t-tests or Wilcoxon rank-sum tests, and values were compared using the chi-square test. All statistical analyses were performed using IBM SPSS Statistics version 25.0 (IBM Corp., Armonk, N.Y., USA). All analyses were two tailed, with a significance level of 0.05.

## 3 Results

**Figure 1:**
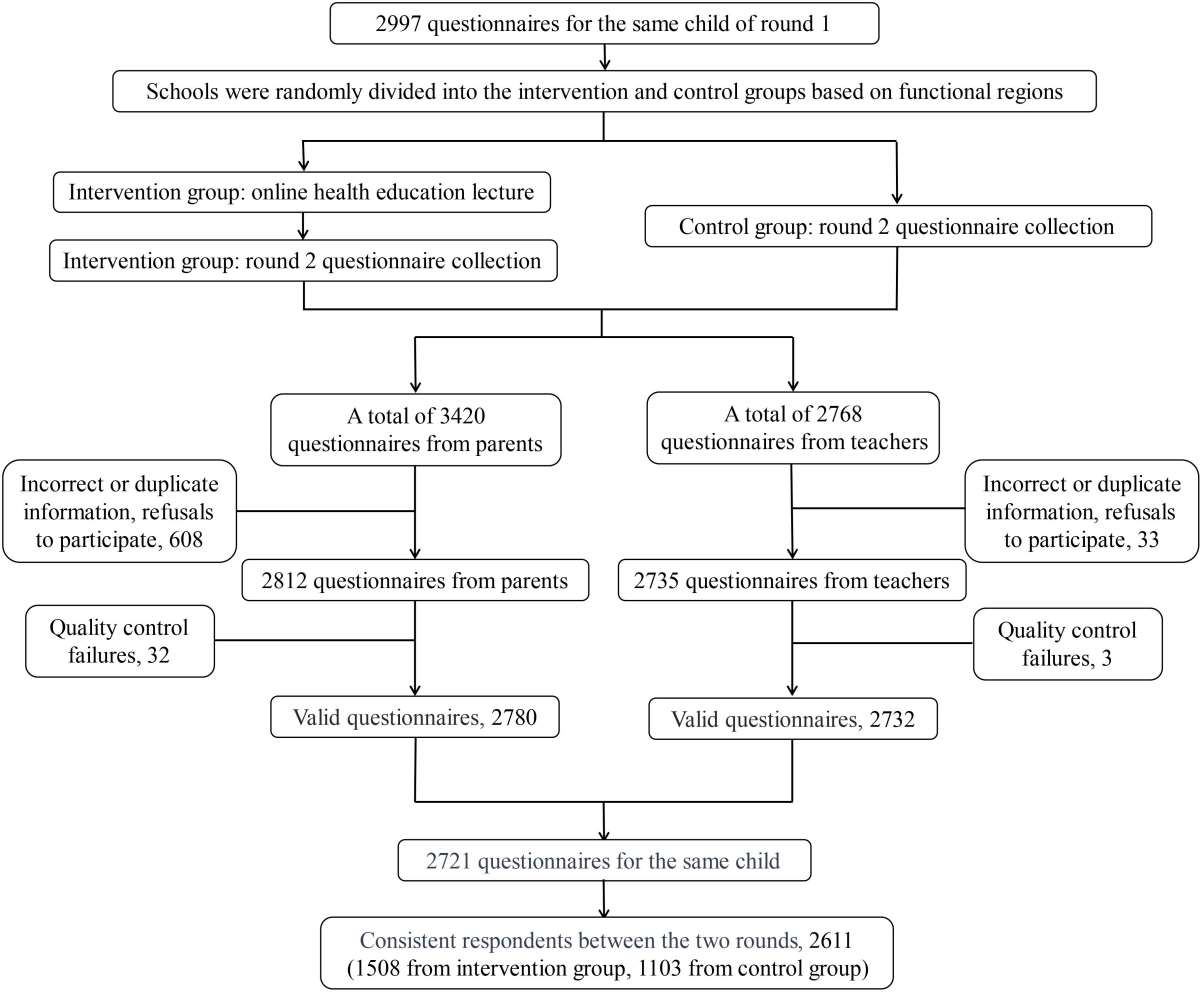
Questionnaire collection.

In the first round, 3780 valid parent questionnaires and 3542 valid teacher questionnaires were collected. Among these, there were 2997 questionnaires where both parents and teachers evaluated the same child. In the second round, 3420 parent questionnaires and 2768 teacher questionnaires were obtained. After excluding questionnaires with incorrect or duplicate information, refusals to participate, quality control failures, and inconsistent respondents between the two rounds, 2611 parent and teacher questionnaires were included, with 1508 questionnaires in the intervention group and 1103 questionnaires in the control group. Approximately 87% (2611/2997) of parents and teachers continued to participate in the second round of data collection (**Figure 1**). **Tables 1A**, **B** present the baseline demographic characteristics and measures of the 2611 children. Approximately 51% of the children were boys, and the mean age was 9.79 years (SD=1.15 years). Of the primary caregivers, 82% were parents, and 67% of the questionnaire respondents had a high school diploma or higher education. Approximately 77% of the teachers had not received any ADHD-related training in the past. The detailed baseline demographic characteristics of the four functional regions are shown in **Supplementary Table S1**.

**Table 1A:** Teacher characteristics.

	Number of teachers	Corresponding number of students
Years of teaching experience, years		
0-9	39 (30.7%)	789 (30.2%)
10-19	25 (19.7%)	503 (19.3%)
20-29	48 (37.8%)	994 (38.1%)
≥30	15 (11.8%)	325 (12.4%)
Number of past ADHD-related training sessions		
Never	97 (76.4%)	2010 (77.0%)
1	12 (9.4%)	232 (8.9%)
2-3	14 (11.0%)	276 (10.6%)
≥4	4 (3.2%)	93 (3.6%)
Total	127	2611

**Table 1B:** Baseline demographic characteristics and measures.

	Total (n=2611)	Intervention (n=1508)	Control (n=1103)	χ²/t (*p*)
Sex				0.295 (0.587)
Male	1330 (50.9%)	775 (51.4%)	555 (50.3%)	
Age (years), mean (SD)	9.79 (1.15)	9.81 (1.15)	9.77 (1.16)	0.775 (0.438)
Primary caregivers				17.646 (<0.001*)
Parents	2146 (82.2%)	1280 (84.9%)	866 (78.5%)	
Others	465 (17.8%)	228 (15.1%)	237 (21.5%)	
Questionnaire respondents				11.987 (0.001*)
Parents	2515 (96.3%)	1469 (97.4%)	1046 (94.8%)	
Others	96 (3.7%)	39 (2.6%)	57 (5.2%)	
Educational levels of respondents				76.668 (<0.001*)
Junior high school or below	849 (32.5%)	396 (26.3%)	453 (41.1%)	
High school or technical secondary school	899 (34.4%)	530 (35.1%)	369 (33.5%)	
College or above	863 (33.1%)	582 (38.6%)	281 (25.5%)	
Length of teaching experience, years				555.725 (<0.001*)
0-9	789 (30.2%)	705 (46.8%)	84 (7.6%)	
10-19	501 (19.2%)	161 (10.7%)	340 (30.8%)	
20-29	996 (38.1%)	538 (35.7%)	458 (41.5%)	
≥30	325 (12.4%)	104 (6.9%)	221 (20.0%)	
Number of past ADHD-related training sessions of teachers				120.569 (<0.001*)
Never	2010 (77.0%)	1079 (71.6%)	931 (84.4%)	
1	232 (8.9%)	135 (9.0%)	97 (8.8%)	
2-3	276 (10.6%)	244 (16.2%)	32 (2.9%)	
≥4	93 (3.6%)	50 (3.3%)	43 (3.9%)	
Positive screening rate of ADHD–parents, round 1	96 (3.7%)	47 (3.1%)	49 (4.4%)	3.161 (0.075)
I+H^a^ scores–parents, round 1	11.19 (6.77)	11.60 (6.70)	10.61 (6.84)	3.378 (<0.001*)
I scores–parents, round 1	7.14 (3.94)	7.42 (3.88)	6.75 (3.99)	4.354 (<0.001*)
H scores–parents, round 1	4.05 (3.44)	4.18 (3.39)	3.86 (3.49)	2.319 (0.020*)
Positive screening rate of ADHD–teachers, round 1	88 (3.4%)	56 (3.7%)	32 (2.9%)	1.291 (0.256)
I+H scores–teachers, round 1	6.82 (7.76)	6.64 (8.05)	7.07 (7.35)	-1.389 (0.165)
I scores–teachers, round 1	4.64 (4.74)	4.58 (5.00)	4.72 (4.37)	-0.767 (0.443)
H scores–teachers, round 1	2.19 (3.65)	2.06 (3.71)	2.35 (3.57)	-1.988 (0.047*)

a I, inattention dimension; H, hyperactivity and impulsivity dimension.

Statistical analysis: Chi-square test or t-test. Statistical significance is indicated by * and bold values (p < 0.05).

Regarding the positive screening rate of ADHD among parents (**Table 2**), the educational intervention did not have an impact. However, there was a difference in the attention deficit symptom scores between the intervention and control groups in the second round of parent questionnaires. The intervention group had higher attention deficit scores than the control group (β 0.42, [95% CI: 0.02 to 0.82], p=0.040). Boys had higher ADHD symptom scores than girls (combined attention deficit and hyperactivity-impulsivity score: β 1.11, [95% CI: 0.71 to 1.51], p<0.001; attention deficit score: β 0.57, [95% CI: 0.33 to 0.81], p<0.001; hyperactivity-impulsivity score: β 0.63, [95% CI: 0.42 to 0.84], p<0.001). When the primary caregiver was a parent, the ADHD symptom scores were lower (combined attention deficit and hyperactivity-impulsivity score: β -0.61, [95% CI: -1.13 to -0.09], p=0.022; attention deficit score: β -0.32, [95% CI: -0.64 to -0.01], p=0.047; hyperactivity-impulsivity score: β -0.30, [95% CI: -0.57 to -0.03], p=0.029). When the educational level of the questionnaire respondent was junior high school or below, the positive screening rate of ADHD (β 0.49, [95% CI: 0.02 to 0.96], p=0.039) and symptom scores were higher (combined attention deficit and hyperactivity-impulsivity score: β 0.60, [95% CI: 0.09 to 1.11], p=0.022; hyperactivity-impulsivity score: β 0.39, [95% CI: 0.12 to 0.65], p=0.004). After excluding the parents of children with ADHD, we obtained similar results (**Supplementary Table S2**).

**Table 2:** Relationship between ADHD screening positivity and ADHD symptom scores with intervention and covariates among parents.

	ADHD screening positivity			I+H scores			I scores			H scores		
	Adjusted Beta (β)	95% CI	*p*-Value	Adjusted Beta (β)	95% CI	*p*-Value	Adjusted Beta (β)	95% CI	*p*-Value	Adjusted Beta (β)	95% CI	*p*-Value
**Intercept (SE)**	-3.78 (0.36)			3.66 (0.42)			2.81 (0.25)			1.25 (0.23)		
Group												
Intervention	-0.37	-0.94 to 0.21	0.208	0.48	-0.26 to 1.23	0.201	0.42	0.02 to 0.82	0.040*	0.09	-0.36 to 0.55	0.695
Control	Reference			Reference			Reference			Reference		
Sex												
Male	0.10	-0.27 to 0.46	0.602	1.11	0.71 to 1.51	<0.001*	0.57	0.33 to 0.81	<0.001*	0.63	0.42 to 0.84	<0.001*
Female	Reference			Reference			Reference			Reference		
Primary caregivers												
Parents	-0.32	-0.74 to 0.10	0.139	-0.61	-1.13 to -0.09	0.022*	-0.32	-0.64 to -0.01	0.047*	-0.30	-0.57 to -0.03	0.029*
Others	Reference			Reference			Reference			Reference		
Educational levels of respondents												
Junior high school or below	0.49	0.02 to 0.96	0.039*	0.60	0.09 to 1.11	0.022*	0.24	-0.07 to 0.55	0.128	0.39	0.12 to 0.65	0.004*
High school or technical secondary school	0.24	-0.24 to 0.72	0.324	0.17	-0.32 to 0.65	0.507	-0.07	-0.37 to 0.23	0.635	0.26	0.00 to 0.51	0.048*
College or above	Reference			Reference			Reference			Reference		
**Scores in round 1^a^**	0.08	0.06 to 0.10	<0.001*	0.58	0.55 to 0.61	<0.001*	0.53	0.50 to 0.57	<0.001*	0.54	0.51 to 0.57	<0.001*

a When calculating the ADHD screening positivity and I+H scores, scores in round 1 mean the I+H scores in round 1; When calculating the I scores, scores in round 1 mean the I scores in round 1; When calculating the H scores, scores in round 1 means the H scores in round 1.

Statistical analysis: Generalized linear mixed model. Statistical significance is indicated by * and bold values (p < 0.05).

In the analysis of the positivity rate of ADHD screening among teachers (**Table 3**), educational intervention did not have an impact on both the rate and symptom scores. The positive screening rates of ADHD (β 0.47, [95% CI: 0.05 to 0.90], p=0.027) and symptom scores (combined attention deficit and hyperactivity-impulsivity score: β 1.75, [95% CI: 1.25 to 2.25], p<0.001; attention deficit score: β 0.89, [95% CI: 0.59 to 1.19], p<0.001; hyperactivity-impulsivity score: β 0.92, [95% CI: 0.68 to 1.17], p<0.001) was higher in boys than in girls. When teachers had 10-19 years of experience, both the positive screening rate of ADHD (β 1.26, [95% CI: 0.38 to 2.14], p=0.005) and symptom scores (combined attention deficit and hyperactivity-impulsivity score: β 2.60, [95% CI: 1.55 to 3.65], p<0.001; attention deficit score: β 1.38, [95% CI: 0.74 to 2.02], p<0.001; hyperactivity-impulsivity score: β 1.16, [95% CI: 0.65 to 1.67], p<0.001) were higher. The number of ADHD-related trainings did not have a significant impact on the outcomes. After excluding those teachers who self-reported having sufficient knowledge of ADHD at baseline based on a subjective self-assessment question (‘How well do you think you understand ADHD?’), the results remained similar (**Supplementary Table S3**).

**Table 3:** Relationship between ADHD screening positivity and ADHD symptom scores with intervention and covariates among teachers.

	ADHD screening positivity			I+H scores			I scores			H scores		
	Adjusted Beta (β)	95% CI	*p*-Value	Adjusted Beta (β)	95% CI	*p*-Value	Adjusted Beta (β)	95% CI	*p*-Value	Adjusted Beta (β)	95% CI	*p*-Value
**Intercept (SE)**	-7.02 (1.18)			0.58 (0.95)			0.22 (0.59)			0.44 (0.45)		
Group												
Intervention	0.53	-0.52 to 1.58	0.338	0.20	-1.21 to 1.60	0.785	0.08	-0.80 to 0.95	0.862	0.10	-0.55 to 0.76	0.755
Control	Reference			Reference			Reference			Reference		
Sex												
Male	0.47	0.05 to 0.90	0.027*	1.75	1.25 to 2.25	<0.001*	0.89	0.59 to 1.19	<0.001*	0.92	0.68 to 1.17	<0.001*
Female	Reference			Reference			Reference			Reference		
Length of teaching experience, years												
0-9	0.42	-0.50 to 1.34	0.371	1.06	0.04 to 2.08	0.042*	0.55	-0.08 to 1.17	0.087	0.48	-0.02 to 0.97	0.059
10-19	1.26	0.38 to 2.14	0.005*	2.60	1.55 to 3.64	<0.001*	1.38	0.74 to 2.02	<0.001*	1.16	0.65 to 1.67	<0.001*
20-29	0.94	0.15 to 1.73	0.020*	1.04	0.18 to 1.91	0.018*	0.55	0.02 to 1.07	0.043*	0.44	0.02 to 0.86	0.041*
≥30	Reference			Reference			Reference			Reference		
Number of past ADHD-related training sessions												
Never	1.52	-0.57 to 3.61	0.153	0.34	-1.12 to 1.81	0.647	0.63	-0.27 to 1.53	0.168	-0.30	-1.01 to 0.41	0.405
1	1.77	-0.39 to 3.94	0.108	0.51	-1.14 to 2.15	0.547	0.72	-0.28 to 1.73	0.159	-0.18	-0.98 to 0.62	0.660
2-3	0.88	-1.33 to 3.01	0.434	0.06	-1.66 to 1.78	0.949	0.69	-0.36 to 1.75	0.197	-0.63	-1.47 to 0.20	0.
≥4	Reference			Reference			Reference			Reference		
**Scores in round 1**	0.11	0.09 to 0.13	<0.001*	0.55	0.52 to 0.58	<0.001*	0.57	0.54 to 0.60	<0.001*	0.49	0.45 to 0.52	<0.001*

Statistical analysis: Generalized linear mixed model. Statistical significance is indicated by * and bold values (p < 0.05).

**Table 4** presents the positivity rates of ADHD screening before and after the educational intervention for parents and teachers. Although there was a significant difference in the positivity rate of ADHD screening within the intervention group before and after the intervention (p=0.046 for parents and p=0.029 for teachers), there was no significant difference between the control and intervention groups. Logistic regression analysis adjusted for baseline levels showed that the educational intervention did not have a significant impact on outcomes. However, in absolute terms, the number of children with positive ADHD screening results was higher in the second round than in the first round in both the intervention and control groups.

**Table 4:** Within-group comparisons of positive rate of ADHD screening among parents and teachers.

	Pre-intervention	Post-intervention	χ²	*p*-Value	OR^a^ (95% CI)	*p*-Value
**Parents**					1.22 (0.79 to 1.89)	0.377
Intervention	47 (3.1%)	68 (4.5%)	3.987	0.046*		
Control	49 (4.4%)	67 (6.1%)	2.948	0.086		
**Teachers**					0.83 (0.56 to 1.24)	0.360
Intervention	56 (3.7%)	81 (5.4%)	4.779	0.029*		
Control	32 (2.9%)	47 (4.3%)	2.954	0.086		

a Logistic regression adjusted for baseline levels.

Statistical analysis: Chi-square test and Logistic regression model. Statistical significance is indicated by * and bold values (p < 0.05).

A total of 907 parents provided specific feedback to the question “Do you have any other comments or suggestions regarding this lecture?”, which was further categorized into positive feedback (e.g., “very good,” “hope for more promotion”), neutral feedback (questioning the questionnaire items, expressing doubts about the child’s condition, reporting a lack of ADHD symptoms in their child), and negative feedback (e.g., “no meaning,” “confusing”). A total of 87.1% of the parents provided positive feedback, 9.3% had a neutral attitude, and only 3.6% provided negative feedback.

With respect to the impact of educational interventions on parental stress among children who screened positive for ADHD in the first round, 96 children were identified to be ADHD positive in the first-round screening. **Table 5** provides an analysis of the demographic characteristics and baseline levels of the 96 children, showing no statistical differences between the two groups. The educational intervention did not have an impact on parental stress among children who screened positive for ADHD, as shown in **Table 6**.

**Table 5:** Baseline demographic characteristics and measures of the children screened positive for ADHD in the first round.

	Intervention (n=47)	Control (n=49)	χ²/t/Z (*p*)
Sex			2.896 (0.089)
Male	32 (68%)	25 (51%)	
Age, years (SD)	10.24 (1.23)	9.95 (0.94)	1.313 (0.193)
Primary caregivers			2.136 (0.144)
Parents	37 (79%)	32 (65%)	
Others	10 (21%)	17 (35%)	
Educational levels of respondents			2.256 (0.324)
Junior high school or below	17 (36%)	25 (51%)	
High school or technical secondary school	20 (43%)	17 (35%)	
College or above	10 (21%)	7 (14%)	
Scores of CGSQ for round 1 (P25, P75)	37.00 (26.00, 48.00)	32.00 (23.00, 38.50)	-1.697 (0.090)
Scores of ISCGS for round 1 (P25, P75)	11.00 (9.00, 16.00)	10.00 (6.00, 14.00)	-1.580 (0.114)
Scores of ESCGS for round 1 (P25, P75)	7.00 (5.00, 9.00)	6.00 (4.00, 7.00)	-1.657 (0.098)
Scores of OCGS for round 1 (P25, P75)	17.00 (11.00, 24.00)	15.00 (11.00, 19.00)	-1.455 (0.146)

Statistical analysis: Chi-square test or t-test or Wilcoxon rank-sum test.

**Table 6:** Impact of online health education lecture on the parenting stress of parents with ADHD-positive children.

	Intervention (n=47)	Control (n=49)	Z (*p*)
Scores of CGSQ for round 2 (P25, P75)	34.00 (28.00, 47.00)	31.00 (24.00, 40.50)	-1.413 (0.158)
Scores of ISCGS for round 2 (P25, P75)	11.00 (9.00, 15.00)	10.00 (7.00, 15.50)	-0.917 (0.359)
Scores of ESCGS for round 2 (P25, P75)	6.00 (4.00, 8.00)	6.00 (4.00, 7.00)	-0.900 (0.368)
Scores of OCGS for round 2 (P25, P75)	16.00 (14.00, 21.00)	15.00 (11.00, 19.00)	-1.727 (0.084)

Statistical analysis: Wilcoxon rank-sum test.

## 4 Discussion

In this study, the ADHD screening positive rate is lower than the national prevalence of ADHD in China (21). Beyond regional differences, we consider this may be attributed to the implementation of the “Double Reduction” policy initiated by the government in 2021 (22). The policy aims to reduce the total amount and duration of students’ homework and alleviate the burden of extracurricular tutoring. As educational pressure decreases, the anxiety levels of parents and teachers might be partially alleviated, leading to increased tolerance for children’s behavior and consequently a lower detection rate of ADHD-like symptoms. Similarly, Italy, the birthplace of Montessori education, also reports a low ADHD prevalence of 2.9% (23). On the other hand, although the Vanderbilt questionnaires are widely used for ADHD screening (24–26) and assessing ADHD symptom severity (27, 28), the subjectivity involved in the evaluation process and variability in the respondents’ educational backgrounds of respondents may introduce biases in the results from parent and teacher questionnaires. These results reflect only the screening positive rate rather than the actual prevalence of the disorder. Clinical diagnosis of ADHD requires specialists to make comprehensive judgments based on patient history, behavioral observations, and psychological assessments. Furthermore, given the subjectivity inherent in questionnaires, increasing public education for parents and teachers on ADHD-related knowledge may help improve the healthcare-seeking rate for children with ADHD in the future.

After adjusting for confounding factors, the educational lecture did not have a significant effect on the positive screening rates of ADHD reported by parents and teachers. Although the rates within the intervention were significantly different before and after the intervention, these differences were no longer significant after adjusting for baseline levels. To our best knowledge, this study is the first to evaluate the impact of an online health education lecture on the positivity rates of ADHD screening and symptom scores in the general population of parents and teachers. Notably, the positive screening rates of ADHD were higher in the second round than in the first round in both the intervention and control groups. This increase could be attributed to the following reasons.

Firstly, both the intervention and control groups underwent the same intervention of completing the first-round questionnaire, which may have introduced a practice effect. As per a study on knowledge learning, testing is more effective than other strategies for improving learning outcomes (29). Parents and teachers may have become aware of ADHD after completing the first-round questionnaire, and they generally have a vested interest in the question of whether their children have ADHD, which may further enhance their motivation to learn. This could have led to a more accurate understanding or more careful observation of related behavioral issues in children, thereby improving the accuracy of responses in the second-round questionnaire. Second, modern patients increasingly rely on the internet and social media as easily accessible sources of medical information (30). In this study, there was a 6-month interval between the two rounds of questionnaire survey, during which parents and teachers may have obtained ADHD-related information from other channels. Natural neurodevelopmental processes in children over time may also have influenced the final results. For example, from prenatal development to adulthood, the prefrontal cortex continuously develops and matures functionally (31), and it is closely related to executive function (32), which may further impact the core symptoms of ADHD.

Third, theoretically, the greater the intervention frequency and intensity, the greater the magnitude of the behavioral change. Particularly, there is a dose-effect relationship between intervention quantity/intensity and effectiveness. Other parent training interventions for ADHD typically have longer durations and are performed at least eight times (14). In this study, only one 90-minute online lecture was provided, which may have resulted in a weak effect, highlighting the importance of implementing health education interventions in a planned and repeated manner.

Fourth, owing to the coronavirus disease 2019 pandemic, we adopted an online education model instead of traditional face-to-face training. Online education may have suboptimal effectiveness. Although previous studies have compared the effectiveness of online and face-to-face training and suggested that both methods are equally effective and that online training is more readily accepted by parents (28, 33), these studies focused on parents whose children had already been diagnosed with ADHD, a population with a stronger motivation to learn. Furthermore, studies in other fields have shown that both face-to-face and online teaching methods can effectively improve outcomes; however, online training programs have lower adherence rates and overall effectiveness (34). Similarly, our study observed that some parents did not actively participate in the lecture. ADHD is a highly heritable disorder, with an estimated heritability of 80% (35). It is likely that the parents of most children with ADHD also have underlying ADHD symptoms or diagnoses. Moreover, individuals with ADHD benefit more from face-to-face small-group instruction. Therefore, online parental education may not be suitable for the parents of children with ADHD.

Finally, during the health education lecture, we introduced child behavior management techniques. When the parents/teachers in the intervention group completed the questionnaire again 1 month after the intervention, they may have learned and applied the child behavior management techniques discussed in the lecture. This could have resulted in changes in children’s behavior.

Although our results showed that the online health education lecture did not have a significant impact on the positive screening rates of ADHD and the ADHD symptom scores reported by teachers and parents, parents in the intervention group reported higher scores for their children in the inattention subscale. One possible explanation is that hyperactive/impulsive symptoms are more easily observed in daily life, and the intervention may have increased the parents’ focus to their children’s inattention symptoms.

Overall, although the analysis showed no significant impact of the educational intervention on positive screening rates of ADHD, there was a significant increase in the absolute number of children who screened positive for ADHD in the second round of questionnaire survey. Considering the predominantly positive feedback from most parents, we speculate that health communication in any form, whether through educational lectures or questionnaires, can strengthen public awareness of ADHD.

In addition, this study found some significant data. When the primary caregiver of the child was the parent, the ADHD symptom scores were lower; in contrast, when the primary caregiver was a grandparent or others, the ADHD symptom scores were higher. This is consistent with the findings of Tong et al. (36) that showed that parents as primary caregivers may be more beneficial. A study conducted in North Carolina, USA showed that children were at a higher risk of ADHD when their parents had lower levels of education (37). Consistent with these findings, the positive screening rate and symptom score were higher in this study when the educational level of the respondents was junior high school or below. This may be related to genetic background, as the heritability of ADHD is approximately 80% (8, 35). ADHD can lead to lower socioacademic achievement; thus, children with parents with lower education levels may exhibit higher ADHD symptom scores. Gene–environment interactions should also be considered. Children’s ADHD-like behaviors can elicit harsh and discouraging parenting styles, leading to escalating problems and the development of coercive cycles within the family (8).

Furthermore, a teaching experience of 10-19 years was associated with higher positive screening rates of ADHD and symptom scores. This may be related to the career trajectory of teachers, as younger teachers may have a greater focus on teaching, adapting to new roles, and building their identity (38), or they may have less experience with ADHD. Teachers with a teaching experience of 20-29 years may experience higher levels of professional burnout (39), leading to a negative, callous, and detached attitude toward students (40), which could result in increased tolerance of children’s behavior. Teachers with a teaching experience of ≥30 years may experience fatigue, leading to increased exhaustion in their work (41) and reduced attention to children’s behavior. Our data showed that approximately 76% of teachers in Chongqing had never received any training related to ADHD. The rate is lower in Shanghai at 38% (42), indicating disparities between regions with different levels of development. This findings highlights that teacher training should be enhanced in economically underdeveloped regions. The online health education lecture did not significantly improve the parenting stress of the parents of children who screened positive for ADHD. As discussed above, this may be due to the short duration and online format of the lecture.

Thus, this study provides valuable insights. First, when evaluating health communication or health promotion projects, it is possible to forgo a control group and only compare changes in health-related behaviors, self-efficacy, values, and other indicators before and after the intervention using a pre-post design (43). However, our study included a control group and emphasized the potential influence of questionnaire completion, which could attract the attention of researchers conducting similar study designs. Second, we emphasize that health communication with the public is always beneficial regardless of the method used, and professionals should educate the general public through diverse methods. Third, when dealing with diseases similar to ADHD that require parental or teacher assistance in assessment, the personal characteristics of the evaluators, such as parents’ education levels and teachers’ years of experience, need to be considered. Concurrently, questionnaire assessments are subjective, and ADHD diagnoses should not rely on questionnaire results.

Our study has some limitations. First, the intervention duration was short, and the educational approach was singular. Longer duration and more engaging health communication methods may yield more positive results. Second, although we implemented quality control measures for the questionnaires, we cannot completely rule out the possibility that some parents and teachers provided arbitrary responses. Third, we did not collect data on the socioeconomic status of the families due to privacy concerns. Additionally, ADHD-related knowledge among parents and teachers was not assessed using objective measures (e.g., standardized quizzes or knowledge tests (4)) during the baseline survey. This may have introduced potential biases. Fourth, this study was only conducted in a single area; hence, the findings may not be generalizable to all regions. However, they still have significant value for underdeveloped areas.

## 5 Conclusion

A single session of online health education may help parents recognize ADHD symptoms to some extent. Systematic, repeated, and combined online and offline interventions could be explored in future studies to enhance their effectiveness. Furthermore, parental education level is correlated with the identification of childhood ADHD symptoms, and teachers with 10-19 years of experience exhibit higher attention scores and a stronger ability to identify ADHD symptoms in children. The training regarding childhood ADHD should be strengthened for both parents and teachers. Such training programs should consider the cultural background of caregivers, educators’ teaching experience, and the frequency of their participation in relevant training.

## Data Availability

The datasets presented in this study can be found in online repositories. The names of the repository/repositories and accession number(s) can be found below: Mendeley data. DOI: 10.17632/bvv8fp46v3.1.
