# Supplementary material for: Impact of online health education on attention-deficit/hyperactivity disorder screening results and parenting stress among school-aged children
Source: Front Psychiatry. 2025 Jan 31;16:1522263. doi: 10.3389/fpsyt.2025.1522263 (PMC11825811; doi:10.3389/fpsyt.2025.1522263)
Supplement: Supplementary file 1 [file DataSheet1.pdf]

Table s1. Baseline demographic characteristics among 4 functional regions

	**Intervention (n=1508)**		**Control(n=1103)**		**χ²/F' (*p*)**
	**Central Urban Area**	**Northeastern District**	**Main Urban District**	**Southeastern District**	
Sex					
Male	370 (50.5%)	405 (52.2%)	338 (49.3%)	217 (52.0%)	1.497 (0.683)
Age (years), mean (SD)	9.84 (1.09)	9.78 (1.20)	9.89 (1.12)	9.59 (1.20)	5.927 (**0.001***)
Primary caregivers					21.863 (**<0.001***)
Parents	635 (86.7%)	645 (83.1%)	533 (77.7%)	333 (79.9%)	
Others	97 (13.3%)	131 (16.9%)	153 (22.3%)	84 (20.1%)	
Questionnaire respondents					22.680 (**<0.001***)
Parents	725 (99.0%)	744 (95.9%)	651 (94.9%)	395 (94.7%)	
Others	7 (1.0%)	32 (4.1%)	35 (5.1%)	22 (5.3%)	
Educational levels of respondents					97.338 (**<0.001***)
Junior high school or below	161 (22.0%)	235 (30.3%)	261 (38.0%)	192 (46.0%)	
High school or technical secondary school	282 (38.5%)	248 (32.0%)	238 (34.7%)	131 (31.4%)	
College or above	289 (39.5%)	293 (37.8%)	187 (27.3%)	94 (22.5%)	
Length of teaching experience, years					843.191 (**<0.001***)
0-9	229 (31.3%)	476 (61.3%)	62 (9.0%)	22 (5.3%)	
10-19	76 (10.4%)	85 (11.0%)	159 (23.2%)	181 (43.4%)	
20-29	371 (50.7%)	167 (21.5%)	293 (42.7%)	165 (39.6%)	
⩾30	56 (7.7%)	48 (6.2%)	172 (25.1%)	49 (11.8%)	
Number of past ADHD-related training sessions of teachers					337.273 (**<0.001***)
Never	444 (60.7%)	635 (81.8%)	533 (77.7%)	398 (95.4%)	
1	63 (8.6%)	72 (9.3%)	84 (12.2%)	13 (3.1%)	
2-3	175 (23.9%)	69 (8.9%)	26 (3.8%)	6 (1.4%)	
⩾4	50 (6.8%)	0 (0%)	43 (6.3%)	0 (0%)	

Table s2. After excluding the parents of children with ADHD, relationship between ADHD screening positivity and ADHD symptom scores with intervention and covariates among parents

	ADHD screening positivity		I+H scores		I scores		H scores	
	Adjusted Beta (β)	*p*-Value	Adjusted Beta (β)	*p*-Value	Adjusted Beta (β)	*p*-Value	Adjusted Beta (β)	*p*-Value
**Intercept**	-3.66		3.75		2.80		1.32	
**Group**								
Intervention	-0.07	0.730	0.40	0.397	0.40	**0.049***	0.06	0.841
Control	Reference		Reference		Reference		Reference	
**Sex**								
Male	0.06	0.742	1.09	**<0.001***	0.55	**<0.001***	0.62	**<0.001***
Female	Reference		Reference		Reference		Reference	
**Primary caregivers**								
Parents	-0.16	0.499	-0.67	**0.013***	-0.35	**0.035***	-0.34	**0.016***
Others	Reference		Reference		Reference		Reference	
**Educational levels of respondents**								
Junior high school or below	0.14	0.543	0.60	**0.022***	0.24	0.128	0.40	**0.003***
High school or technical secondary school	0.06	0.805	0.16	0.526	-0.07	0.659	0.25	0.059
College or above	Reference		Reference		Reference		Reference	
**Scores in round 1**	0.06	**<0.001***	0.58	**<0.001***	0.54	**<0.001***	0.54	**<0.001***

Table s3. After excluding those teachers who had sufficient knowledge of ADHD at baseline, relationship between ADHD screening positivity and ADHD symptom scores with intervention and covariates among teachers

	ADHD screening positivity		I+H scores		I scores		H scores	
	Adjusted Beta (β)	*p*-Value	Adjusted Beta (β)	*p*-Value	Adjusted Beta (β)	*p*-Value	Adjusted Beta (β)	*p*-Value
**Intercept**	-6.95		1.72		0.94		0.87	
**Group**								
Intervention	0.56	0.337	-0.01	0.988	-0.06	0.914	0.04	0.911
Control	Reference		Reference		Reference		Reference	
**Sex**								
Male	0.57	**0.015***	1.76	**<0.001***	0.89	**<0.001***	0.93	**<0.001***
Female	Reference		Reference		Reference		Reference	
**Length of teaching experience, years**								
0-9	0.225	0.635	0.10	0.854	-0.04	0.910	0.09	0.728
10-19	0.957	**0.039***	1.46	**0.007***	0.685	**0.040***	0.73	**0.006***
20-29	0.608	0.139	-0.02	0.964	-0.10	0.705	0.02	0.928
⩾30	Reference		Reference		Reference		Reference	
**Number of past ADHD-related training sessions**								
Never	1.48	0.165	0.41	0.578	0.67	0.139	-0.28	0.436
1	1.60	0.152	0.57	0.491	0.77	0.131	-0.16	0.689
2-3	0.83	0.466	-0.03	0.969	0.63	0.238	-0.66	0.117
⩾4	Reference		Reference		Reference		Reference	
**Scores in round 1**	0.11	**<0.001***	0.55	**<0.001***	0.57	**<0.001***	0.49	**<0.001***
